# EfficientMaize: A Lightweight Dataset for Maize Classification on Resource-Constrained Devices

**DOI:** 10.1016/j.dib.2024.110261

**Published:** 2024-03-04

**Authors:** Emmanuel Asante, Obed Appiah, Peter Appiahene, Kwabena Adu

**Affiliations:** Department of Computer Science and Informatics, University of Energy and Natural Resources, Sunyani

**Keywords:** Machine learning, Maize dataset, Image recognition, Machine learning algorithm, Precision agriculture, Convolutional Neural Network

## Abstract

Hyperspectral imaging, combined with deep learning techniques, has been employed to classify maize. However, the implementation of these automated methods often requires substantial processing and computing resources, presenting a significant challenge for deployment on embedded devices due to high GPU power consumption. Access to Ghanaian local maize data for such classification tasks is also extremely difficult in Ghana. To address these challenges, this research aims to create a simple dataset comprising three distinct types of local maize seeds in Ghana. The goal is to facilitate the development of an efficient maize classification tool that minimizes computational costs and reduces human involvement in the process of grading seeds for marketing and production. The dataset is presented in two parts: raw images, consisting of 4,846 images, are categorized into bad and good. Specifically, 2,211 images belong to the bad class, while 2,635 belong to the good class. Augmented images consist of 28,910 images, with 13,250 representing bad data and 15,660 representing good data. All images have been validated by experts from Heritage Seeds Ghana and are freely available for use within the research community.

Specifications Table

**Table:** 

Subject	Applied Machine Learning
Specific subject area	Seed Classification
Data format	Raw, Augmented
Type of data	Maize seed Images
Data collection	The dataset consists of three types of maize seeds, namely Wang Dataa, Sanzal Sima, and Bihilifa, all obtained from Heritage Seeds Ghana. These maize varieties are commonly grown in the northern region of Ghana. At the collection point, Heritage Seeds Ghana manually sorted and labelled the images as either good or bad for each of the three varieties. The images were captured using a 12-megapixel phone camera, and the original jpeg images had varying dimensions. To ensure uniformity and clarity, a blue background was used during the image capture process. Finally, the images were organized into their respective classes of good and bad.
Data source location	University of Energy and Natural Resources P. O. Box 214, Sunyani-Ghana Website: https://www.uenr.edu.gh Heritage Seeds Ghana P. O. Box TL 1596, Tamale Website: HERITAGE SEEDS | Farmer-Based Organizations in Ghana (fbosecretariatghana.com)
Data accessibility	Repository name: Lightweight Dataset for Maize Classification on Resource-Constrained Devices Data identification number: 10.17632/r6vvm5jkh6.2 Direct URL to data: https://data.mendeley.com/datasets/r6vvm5jkh6/2

## 1 Value of the Data

• The dataset comprises a total of 28,910 augmented images and 4846 raw images, encompassing three varieties of maize, each categorized into two classes: good and bad.
• It includes maize seeds suitable for production and those unsuitable for crop yield or planting.
• This dataset proves valuable for developing applications focused on maize classification, detection, and recognition.
• It is particularly beneficial for training, testing, and validating high-quality maize seeds to enhance crop yield and for constructing classification and identification models.
• The dataset serves as a valuable resource for creating a maize classification tool optimized for resource-constrained devices.
• It will facilitate the development of an application dedicated to classifying, identifying, and detecting maize seeds. This application can be utilized by farmers, agricultural extension officers, the Ministry of Food and Agriculture (MoFA), and other relevant agencies.

## 2 Data Description

The bedrock of human existence, and indeed the very survival of humanity, is agriculture. With over 7 billion people already inhabiting the planet, the next few decades will see a further 2.5 billion individuals added to the global population, most of whom will live in cities situated in Asia and Africa [2]. Agriculture plays an incontrovertibly decisive role in the continued existence of humankind. The effective and precise management of agricultural processes and resources will determine the welfare and prosperity of people across the world, securing a sustainable future for generations to come [3]. Together with air and water, sustenance is a fundamental prerequisite for the sustenance of human beings. Nonetheless, the risk of food scarcity looms as agricultural land is usurped by urbanization and the global population burgeons [3]. According to the Food and Agriculture Organization (FAO) of the United Nations, smart Agriculture, also known as precision agriculture, is the use of advanced technologies such as sensors, drones, robotics, and artificial intelligence (AI) to optimize agricultural practices [4] Maximizing crop yield, reducing costs, and maintaining healthy ecosystems are among the primary objectives of agricultural production. Maize (Zea mays L.) is a crucial crop that is produced and consumed by most farming households in Ghana, according to the Ministry of Food and Agriculture [5]. According to Heritage Seeds Ghana, accurate classification of the maize seed is essential for cultivation and marketing purposes. Hyperspectral imaging with deep learning has been employed for maize classification, ensuring quality production, seed quality control, and impurity identification. Zhou et al. for instance utilized LesNet-5 to improve maize classification[6]. In a study by Sang et al. [7], they introduced a lightweight CNN architecture based on the MobileNetV2 accelerator for real-time seed sorting. The MobileNetV2 [8] model showed high accuracy, reaching 97.91% for red kidney bean classification and 96.50% for maize seed classification, demonstrating its effectiveness in accurate and efficient real-time seed sorting. Xu et al. [9] presented an enhanced CNN architecture that combined deep learning and machine vision techniques for classifying maize seeds of different varieties. The improved architecture achieved an impressive average classification accuracy of 99.70% on a dataset of 8080 maize seeds from five varieties. Wang and Su [10] conducted a study that presented a detailed exploration of innovative Convolutional Neural Network (CNN) models integrated with computer vision (CV) techniques for detecting phenotypes in grain crops. Qiu et al. [11] investigated using hyperspectral imaging with Convolutional Neural Network (CNN) for rice seed variety identification. The study compared CNN's performance against K-Nearest Neighbors (KNN) and Support Vector Machine (SVM) models in the same task. In the research conducted by G. Qiu et al. in 2019, the application of Fourier Transform Near-Infrared (FT-NIR) spectroscopy combined with discriminant analyses was explored as a rapid and nondestructive method for classifying sweet corn seed cultivars [12]. Bai et al. [13] studied the classification of eight maize seed varieties, including common and silage maize, using Support Vector Machine (SVM) and Radial Basis Function Neural Network (RBFNN) models. The models achieved high accuracies, with over 86% for the direct classification of all eight varieties and over 88% for distinguishing between common and silage maize seeds. The classification accuracy for silage maize seeds surpassed 98%, while for common maize seeds, it exceeded 97%. Despite the recent improvements, Maize seeds are still manually graded and sorted, and this practice continues to this day in Ghana. The manual grading and sorting of maize seeds are a labor-intensive process and require skilled workers who can differentiate between good and bad seeds [14]. The data source, number of variables, variable types and a sample of raw images of the various datasets utilized in the works explored have been summarized in Table 1.

**Table 1:** Comparison of previous works.

Data Source	Authors	Number of Varieties	Variety Types	Raw Image (s)
Normal maize seeds from Beijing Xinfadi farmer's market, China	[6]	6	Jing- nongke728 (JNK728), Shenyu21 (SY21), Xianyou666 (XY666), Jun- dan20 (JD20), Dongmin21 (DM21), and Zhengdan958 (ZD958)	
Normal and silage maize from Yunnan Quchen Seed Co. Ltd., Yunnan, China	[13]	8	Common maize Datian387 (DT387), Quchen8 (QC8), Quchen11 (QC11), and Quchen13 (QC13). Silage maize Quchen9 (QC9), Quchen19 (QC19), Quchen29 (QC29) and Quchen513 (QC513)	
National Seed Breeding Base in Hainan, China	[9]	5	BaoQiu, KouXian, LiaoGe, ShanCu, and XinNuo	
Haploid and diploid corn seed dataset published by Sakaria Maize Research Institute, Turkey	[7]	Unknown	Unknown	
Heritage Seeds, Ghana	[1]	3	Sanzal sima, Bhihilifa, Wang Dataa	Refer to Figure 2(a) (b)

**Figure 1:**
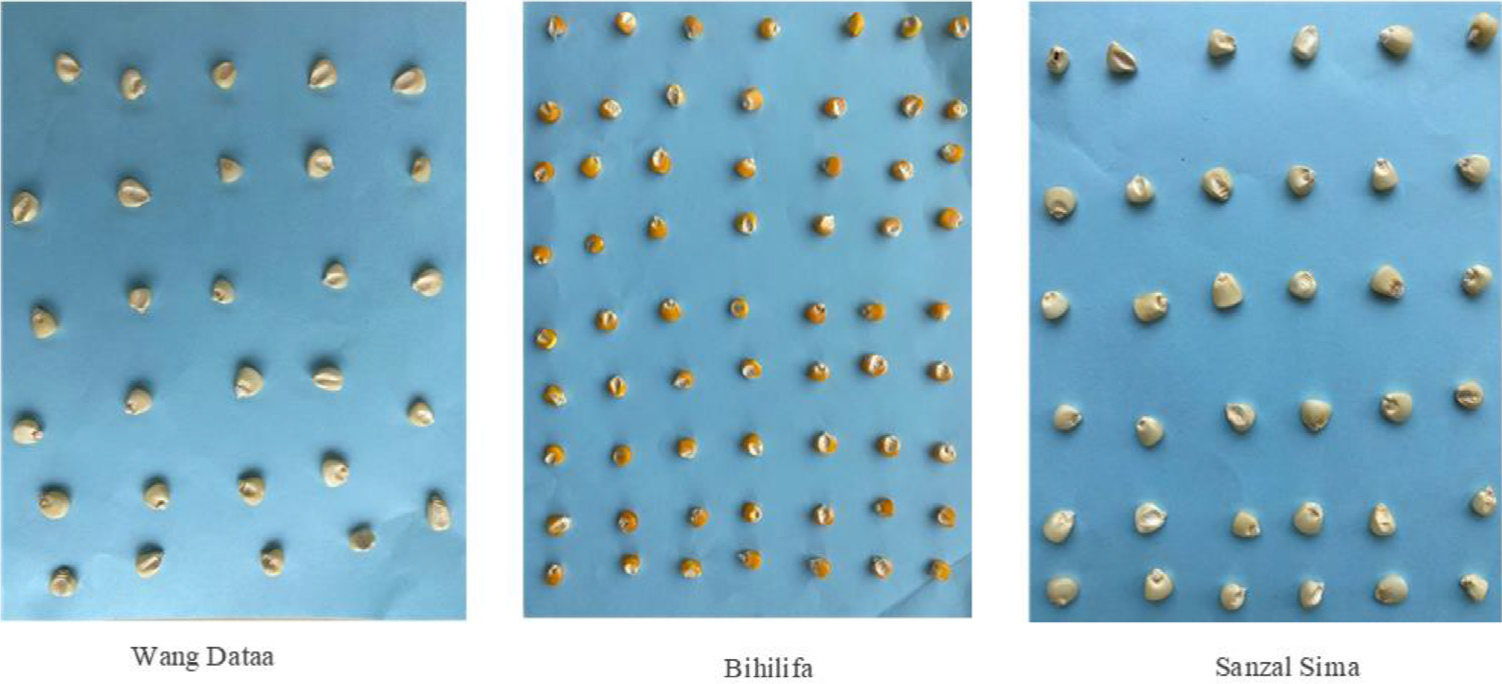
Grouped capture of maize seeds for the three varieties.

The dataset associated with this work contains raw (*4,846 images*) and Augmented (*28,910 images*) color images with blue background and two classes. The raw images are varied in sizes of 76 × 76, 73 × 73, 102 × 102, 75 × 75, 104 × 104, 64 × 64 etc. The augmented images are compressed to a size of 128 × 128. The parameters used to augment the raw data have been outlined in Table 3. This paper provides a dataset for deep learning classification, detection, and recognition tasks for single and multiple models. Having high-resolution and perfect lighting images can increase models’ performance but increase computational costs. Yet the optimal selection of image resolution and model definition can increase neural network performance for various image processing tasks. The raw and augmented images are presented in two folders, good and bad. The raw images can be downloaded as a 6.72 MB file EfficientMaize. The Augmented dataset can be downloaded as a 51.1 MB file Augmented EfficientMaize. The bad folder contains images of bad maize seeds while the good contains images of quality maize seeds for production and feeding. Figure 1 depicts the images captured before cropping and labeling.

**Figure 2:**
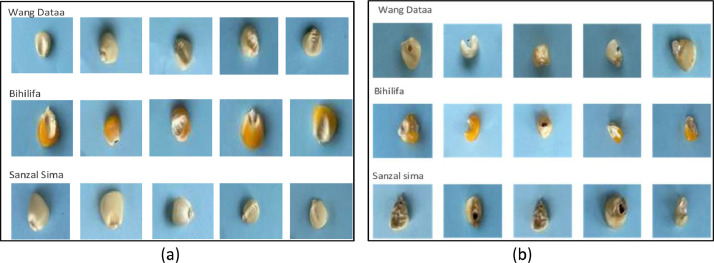
Individual Seeds after cropping and labelling as good and bad.

**Figure 3:**
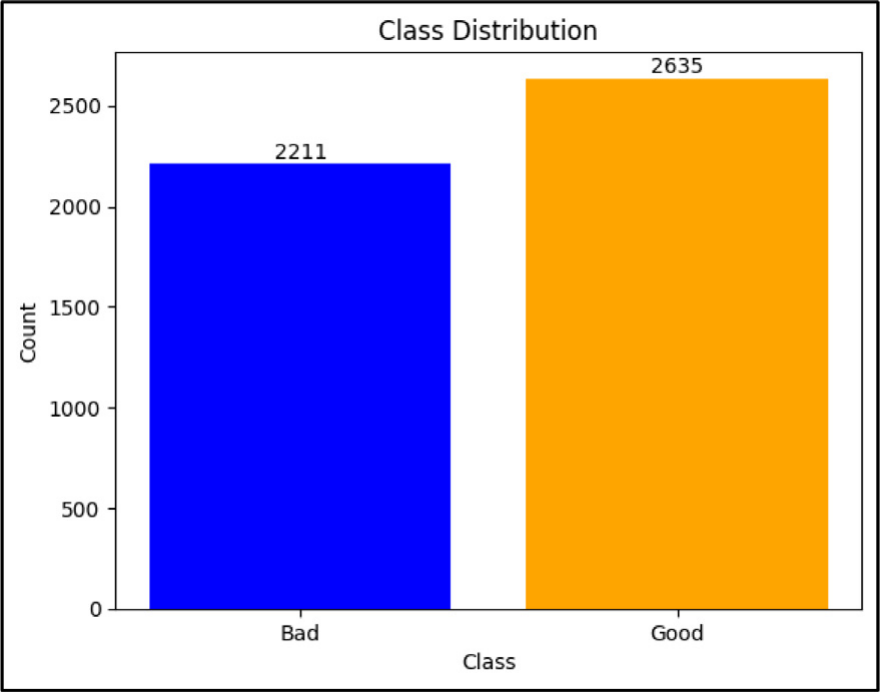
Class distribution for the raw dataset.

**Figure 4:**
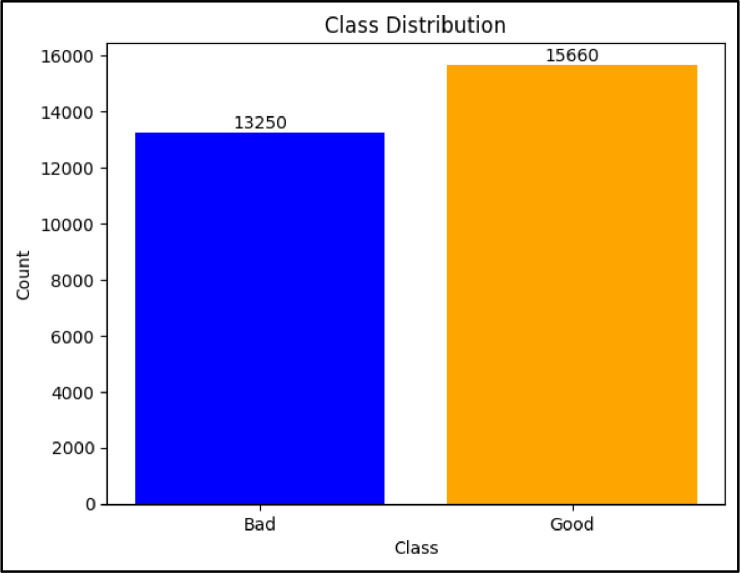
Class distribution for the augmented dataset.

Table 2 shows the specification of the camera used in capturing the dataset. In order to train a deep learning model to identify images under resource constraint conditions, low-quality images are used as input. To achieve this, a 12-megapixel camera was employed to capture the images. To prepare the images for further processing, they were resized to a standard size of 640 × 480 pixels using a Python script. We defined the Image extractor tool to crop the grouped images into their respective classes. The resulting cropped images were deemed satisfactory in terms of quality and suitability for model building. The next step involves labeling the images based on their quality, distinguishing between good and bad seeds. This labeling process was accomplished using the Image Extractor tool. The labeled images were then organized into separate datasets for the good and bad seed categories. The seeds before the image capturing were already grouped by experts from heritage seeds Ghana as good and bad seeds. And these experts also verified the images and labeled them over 2 weeks to ensure their validity and accuracy. Figure 2(a) and (b) depict the labeled and grouped images into their respective classes as good and bad. The total distribution of the raw and augmented data is depicted in Figures 3 and 4 respectively. Table 4 provides details on images such as total images in each class, image size, background, etc.

**Table 2:** Camera specification table.

Specification	Description
**Model**	12 MP iPhone 11 Pro max
**Capacity**	64GB
**Display**	Super Retina XDR display HDR display 2688‑by-1242‑pixel resolution at 458 ppi True Tone display
**Camera**	Triple 12MP Ultra-Wide, Wide, and Telephoto cameras Ultra-Wide: ƒ/2.4 aperture and 120° field of view. Wide: ƒ/1.8 aperture Telephoto: ƒ/2.0 aperture 2x optical zoom in, 2x optical zoom out; digital zoom up to 10x Portrait Lighting with six effects (Natural, Studio, Contour, Stage, Stage Mono, High-Key Mono) Sapphire crystal lens cover 100% Focus Pixels (Wide) Auto Adjustments Next‑generation Smart HDR for photos Wide color capture for photos and Live Photos Advanced red‑eye correction Auto image stabilization Burst mode. Image formats captured: HEIF and JPEG

**Table 3:** Data Augmentation Parameters.

Parameter	Value	Description
rotation_range	20	Specifies the range (in degrees) within which the images can be randomly rotated.
width_shift_range	0.2	Defines the range (as a fraction of the image width) for randomly shifting the images horizontally.
height_shift_range	0.2	Specifies the range (as a fraction of the image height) for randomly shifting the images vertically.
shear_range	0.2	Controls the maximum shear angle in radians for applying random shear transformations to the images.
fill_mode	nearest	Determines the strategy for filling in any newly created pixels during transformations. It uses the nearest pixel values.
brightness_range	[0.5,1.5]	Defines the range for randomly adjusting the brightness of the images.

**Table 4:** Details of lightweight maize Dataset.

S. N	Crop	Categories	No. of classes	Background	No. of Images	
					Raw Data	Augmented Data
1	Maize	Good	2	Blue	2,635	15,660
		Bad			2,211	13,250
Total					**4,846**	**28,910**

**Figure 5:**
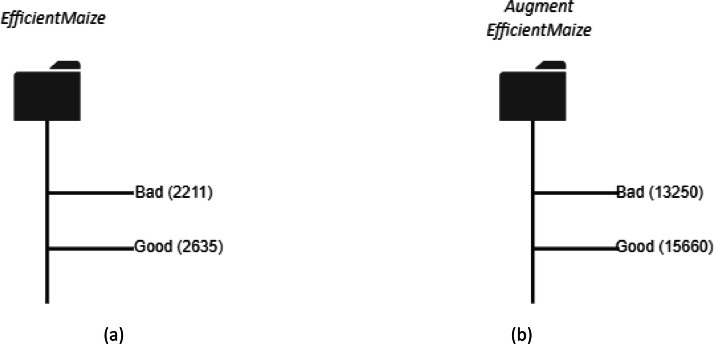
Folder path to EfficientMaize dataset. (a) raw data and (b) augmented data.

Based on the distributions, we can observe that in both the raw data and augmented data, 46% of the total data belongs to the bad class, while the remaining 54% belongs to the good class. This distribution indicates that the dataset is relatively balanced between the two classes, with a slightly higher representation of good maize seeds compared to bad seeds. The directory structure of both raw and augmented data is shown in Figure 5.

## 3 Experimental Design, Materials and Methods

**Figure 6:**
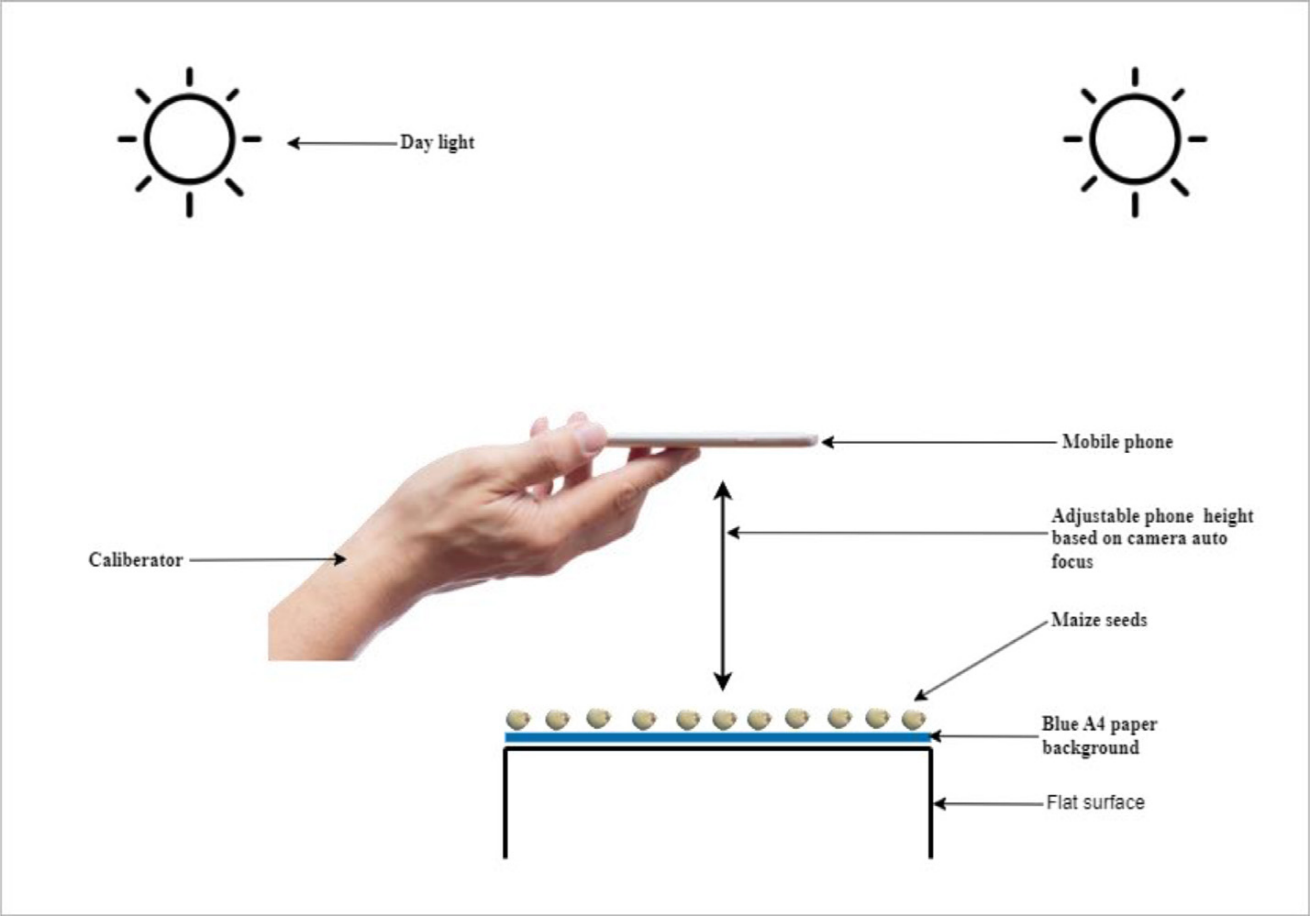
Image Acquisition Setup.

**Figure 7:**
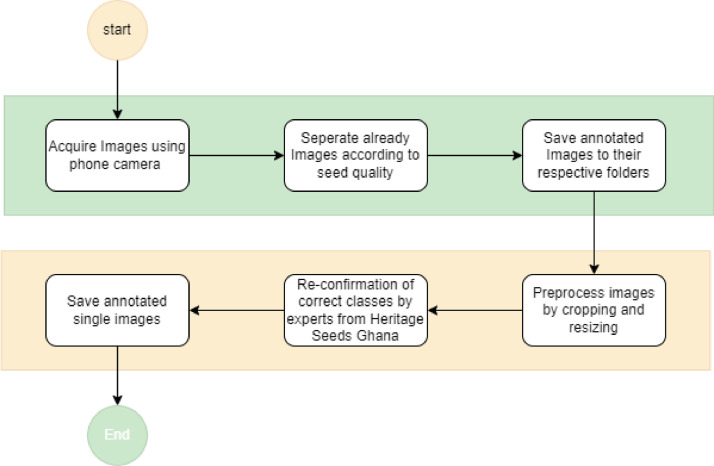
Image acquisition process.

Figure 7 shows the image data acquisition process. The image processing procedure begins with the collection of raw data, which includes three types of commonly planted maize seeds in the northern region of Ghana: Wang Dataa, Sanzal Sima, and Bihilifa, categorized as either good or bad. Images from the raw data were captured using an iPhone 11 Pro Max with a setup as shown in Figure 6. These images were taken in groups on an A4 sheet with a blue background as indicated in Figure 6 and Figure 1 to ensure consistency and clarity during the daytime, without particular attention to lighting conditions. Subsequently, a Python script resized the images to a standard size of 640 by 480 pixels which was required by the image extractor tool for further cropping and labeling. The Image Extractor tool was employed to crop individual images from the grouped ones, and the resulting cropped images were deemed suitable for model building in terms of quality. Next, the images were labeled based on their quality, distinguishing between good and bad seeds. This labeling process involved using the Image Extractor tool, with the assistance of a specialist from Heritage Seeds Ghana. The labeled images were then organized into separate folders for the good and bad seed categories. The good category represented high-quality maize seeds in terms of yield, while the bad category comprised damaged, infected, or low-quality seeds collected from the Headquarters of Heritage Seeds Ghana. The resulting cropped images had varying sizes. Preprocessing steps included image cropping to focus on the regions of interest (ROI). The acquisition of the dataset occurred during the maize harvest season in Ghana, spanning from December 2022 to January 2023. Daily captures were made during daylight hours within this period. Table 5 provides the timeline details of the dataset acquisition process. The folder structure of the images is shown in Figure 5 The dataset consists of only good and bad maize seeds.

**Table 5:** Dataset acquisition timeline.

No.	Step	Duration (weeks)	Activity
1	Data Gathering	2 Weeks	Daily and during daytime capturing of the maize seed images
2	Image Annotation	1 Week	Labeled the 4846 images of good and bad maize images
3	Image Preprocessing	2 Weeks	Cropping the individual seeds images from the grouped images into their respective folders

## Limitations

Different backgrounds would be ideal to test all scenarios since we want to achieve low processing and computing power on the images for the purpose of classification. Notwithstanding, other backgrounds were blurring our images and causing disparity between good and bad seeds making it difficult to identify them from the captured images. The cropping of the images also led to some few images with non-uniform background.

## Ethics Statement

The dataset that supports this work was collected from Heritage Seeds Ghana A consent form to seek approval for the data collection was presented and filled by Mr. Abubakar given their approval to embark on the data collection on their processed maize seeds for this research endeavour.

## CRediT authorship contribution statement

**Emmanuel Asante:** Data curation, Conceptualization, Methodology, Software, Writing – original draft. **Obed Appiah:** Conceptualization, Methodology, Software, Writing – original draft, Supervision, Validation. **Peter Appiahene:** Writing – original draft, Validation, Supervision. **Kwabena Adu:** Writing – original draft, Validation.

## Data Availability

A Lightweight Dataset for Maize Classification on Resource-Constrained Devices (Original data) (Mendeley Data).
